# Non-phonological Strategies in Spelling Development

**DOI:** 10.3389/fpsyg.2020.01071

**Published:** 2020-06-05

**Authors:** Naymé Salas

**Affiliations:** Departament de Didàctica de la Llengua i la Literatura, i de les Ciències Socials, Facultat de Ciències de l’Educació, Universitat Autònoma de Barcelona, Bellaterra, Spain

**Keywords:** spelling, morphology, orthographic constraints, orthographic representations, Catalan

## Abstract

This paper investigated the role that types of knowledge beyond phonology have on spelling development, such as knowledge of morpheme-to-grapheme mappings, of orthographic patterns, and of word-specific orthographic patterns. It is based on the modern view that children do not learn spelling in discrete stages but, rather, they apply different types of strategies from early on. The goals of the paper were threefold: (1) to determine the relative difficulty of different types of non-phonological spelling strategies, (2) to examine the contribution of non-phonological strategies (specifically, morphological, morphophonological, orthographic, and lexical) to conventional spelling scores, and (3) to determine the role of children’s educational level and population type (first- vs. second-language learners) on spelling strategy use. A large sample of 982 children (497 boys), speakers of Catalan (a Romance language similar to Spanish but with a less consistent orthography), participated in the study. They were administered a bespoke dictation task aimed to test their conventional and phonographic accuracy skill, as well as to determine their ability to use different types of non-phonological strategies for the spelling of ambiguous phonemes. Data were analyzed with a series of multigroup, multilevel SEMs. Results showed that (1) children across groups found morphological and lexical strategies harder to apply than orthographic and morphophonological strategies and (2) all types of non-phonological strategies contributed greatly to spelling accuracy scores, even after controlling for children’s phonographic skills. Efficient strategy use increased as a function of schooling level, while second-language learners had a worse performance throughout, but no group showed a specific pattern of results. In conclusion, the paper offers substantial evidence that non-phonological strategies are paramount to learning to spell at least during the early and intermediate elementary school years. It is suggested that the teaching of writing should therefore be multidimensional in nature and target particularly the strategies with which children struggle the most: knowledge of morpho-graphemic mappings and word-specific lexical representations. Theoretical implications are also discussed.

## Introduction

Learning to spell is a process of a phonological nature (Read, 1971; Treiman, 2004). There is substantial evidence, however, that accurate spelling also requires accessing and applying other types of knowledge beyond phonology, such as knowledge of morpheme-to-grapheme mappings (e.g., Nunes et al., 1997a; Pacton and Deacon, 2008) and of orthographic patterns (e.g., Treiman, 1994; Cassar and Treiman, 1997; Pacton et al., 2002; Deacon et al., 2008). This holds true particularly in highly inconsistent orthographies, like English (Borgwaldt et al., 2004; Caravolas et al., 2012), that often need to resolve the ambiguities generated by the multiple alternative spellings for a single phoneme. Nonetheless, users of more consistent orthographies have also been shown to apply non-phonological strategies during spelling (e.g., Defior et al., 2008; Carrillo et al., 2013; Alegría and Carrillo, 2014; Carrillo and Alegría, 2014; Rothe et al., 2014; Marinelli et al., 2017; Angelelli et al., 2018; Zarić et al., 2020). In this paper, we examined the use of non-phonological spelling strategies in Catalan-speaking children in the early and intermediate elementary school years.

### Models of Spelling Development

Early models of spelling were concerned with identifying the various phases in its development. A common trait in stage-like theories of spelling was that the various stages were articulated around the role of phonology. For example, most models distinguished between a pre-phonological phase (i.e., pre-literate), where the nature of the link between language and graphemes is unknown to the child; a phonological phase, where the child has grasped the alphabetic principle (Byrne, 1998) and becomes increasingly more able to represent the phonological structure of words; and a “beyond phonology” phase, in which the child recruits the necessary (non-phonological) knowledge to arrive at the conventional spelling of words (e.g., Gentry, 1978; Henderson and Beers, 1980; Frith, 1985). By collapsing all non-phonological aspects into a single, later stage, these theories did little to accommodate their precise nature and their role in learning to spell.

Currently, the widely accepted view on spelling development is that children are sensitive to non-phonological information right from the start of the learning process and that different spelling strategies overlap throughout development (Rittle-Johnson and Siegler, 1999; Treiman, 2017). What is more, sensitivity to non-phonological aspects of spelling, such as the “outer form” of words, actually precedes phonological processes (Treiman, 2017, p. 4). For example, even pre-phonological spellers develop ideas about the number of graphemes that words have, which are consistent with the average word length in the language to which they are exposed (e.g., Ferreiro and Teberosky, 1979). They have also been found to develop orthographic awareness skills prior to their understanding of the alphabetic principle; for instance, they are sensitive to the positions at which the orthography allows letter doubling and those at which it does not (Cassar and Treiman, 1997).

Children appear to be sensitive to morpho-graphemic regularities in spelling as well (e.g., Nunes et al., 1997a, b; Sénéchal, 2000; Deacon et al., 2008). Treiman and Cassar (1996) showed that young children, aged 5–9 years, were more prone to spelling complex consonant clusters correctly when one of the consonants involved a past tense morpheme (e.g., *passed* > *past*). Although it could be argued that complex spelling strategies, such as morphological knowledge, are mostly applicable in highly inconsistent orthographies, such as French or English, there is evidence of the use of morphological strategies in highly consistent orthographies, such as Spanish (e.g., Defior et al., 2008: Suárez-Coalla et al., 2017), Italian (e.g., Angelelli et al., 2014, 2017), or Finnish (e.g., Lehtonen and Bryant, 2005). Defior et al. (2008), for example, studied the spelling strategies of children who speak a regional dialect of Spanish (Andalusian) in which some consonant endings are not pronounced (e.g., /s/ in coda position). They asked children in grades 1–3 to spell words in two conditions: one in which the final /s/ belonged to a verbal morpheme, as is the case with the <s> in *tienes* “have_2nd person singular” and a control condition in which the /s/ did not have any morphological bearing, as is the case with the <s> in *lunes* “Monday.” Children across grades were more prone to spelling the silent <s> in the morphologically bound condition than the reverse.

In sum, there is abundant evidence that children are sensitive to regularities beyond phonology and that they apply them from very early on. However, only a few studies have addressed the extent to which these strategies are applied successfully at different educational levels. Moreover, a majority of studies on the use of non-phonological spelling strategies has been conducted in English or French, both languages with highly inconsistent phonographic mappings (Borgwaldt et al., 2004; Caravolas et al., 2012), which could arguably make such strategies indispensable, in contrast to more consistent orthographies that could rely on phonological representations to a much larger degree. In this study, we examined the use of non-phonological spelling strategies in early- (grade 2) and intermediate-level (grade 4) speakers of Catalan, a Romance language spoken in Barcelona (Spain), with a semi-transparent orthography (Llauradó and Tolchinsky, 2016).

### Spelling Development in a Second Language

Literacy skills have often been regarded as transferable across the languages spoken by an individual (e.g., Cummins, 1979). However, research on second-language (L2) spelling has reported conflicting findings. On the one hand, L2 learners show low spelling accuracy levels and appear to develop at a slower rate, in comparison with their L1 peers, but their spelling performance appears to be driven by similar skills (Geva et al., 1993; Verhoeven, 2000). Moreover, L2 spelling can be explained, to a great extent, by L1 spelling skills (Sparks et al., 2008), in line with Cummins’ (1979) assertion. Conversely, some studies reported that the skills underlying L1 and L2 spelling differ over time (Jongejan et al., 2007), where at least part of the differences may be related to the characteristics of the L1 writing system (Martin, 2017). Importantly, studies of brain dynamics have shown varying patterns of EEG activity as a function of population type (i.e., L1 vs. L2), especially during the spelling of words that require non-phonological strategies (Weber et al., 2013). In this study, we compared children with (L1) and without (L2) exposure to Catalan, the language of instruction, outside school.

### Assessing Spelling

The analysis of misspellings allows understanding the type of strategies that children apply during spelling. Several schemes have been proposed that have different outcomes and goals. One type of assessment focuses on giving children (partial) credit for misspellings that to some extent reflect the underlying phonological structure of the target words. For example, some schemes evaluate whether children’s written productions represent all phonemes in the target word, using a letter or grapheme that could represent the intended phonemes in some context, even if the resulting production is unconventional, such as writing ^∗^<fait> for *fight* (e.g., Ritchey et al., 2010; Treiman et al., 2019). Other assessment schemes evaluate the degree of phonological proximity of the misspelling to the target word. Caravolas et al. (2001) scored each sound segment and their corresponding grapheme on a 0–4 scale, so that any plausible spelling of a specific sound got the maximum score, and close approximations (e.g., graphemes that represent a phoneme that differs from the target one in a single feature) were scored lower, while omissions were given a score of 0 (Caravolas et al., 2001, p. 758). Phonology-centered assessments of spelling have been particularly insightful when evaluating children in the early stages of learning to spell, when they are expected to use a phonological strategy to spell both known and unknown words. Even most spelling instruction programs recommend beginning with phonologically based strategies [e.g., National Reading Panel, 2000; Alves et al., 2018]. Typically, these assessment schemes do not penalize misspellings for not observing orthographic constraints, since the aim is to determine the extent of children’s phonographic skills.

As reviewed above, there is abundant evidence of children’s early sensitivity to orthographic regularities. Therefore, assessment proposals that focus on evaluating children’s knowledge of orthographic patterns or their familiarity with the “outer-form” of words are highly valuable (Treiman, 2017). Letter-based schemes are characterized by giving children credit for partial success in representing the conventional form of printed words. One of such attempts, for example, consists in awarding points for each two-letter sequence that is accurately (i.e., conventionally) represented, plus points for conventionally represented initial and final letters (Frisby, 2016). This type of schemes is useful to tap into children’s knowledge of orthographic patterns and to test the strength of their orthographic representations in greater detail than a simple correct/incorrect spelling measure.

Other spelling assessment proposals evaluate a combination of phonological and orthographic knowledge. For example, Treiman et al. (2016) assessed children’s spelling considering both phonographic and orthographic skills. Words spelled with all conventional letters received more points than productions that were spelled with phonologically plausible, but unconventional, letters.

Yet other spelling assessment schemes pay attention to children’s use of knowledge sources beyond phonology and orthography (e.g., Masterson and Apel, 2010; Bahr et al., 2012; Lee and Al Otaiba, 2017). Bahr et al. used a framework for assessing spelling rooted on triple word-form theory, which takes into account phonological, orthographic, and morphological strategies, the Phonological, Orthographic, and Morphological Assessment of Spelling (POMAS; Silliman et al., 2006; Bahr et al., 2009, 2012). Investigations using the POMAS scheme classify misspellings as phonological when they omit, add, or replace a letter, so that the resulting word does not preserve the phonological structure of the target word, as in ^∗^<borked> for *worked.* Orthographic errors are those in which the misspelling does not observe orthographic patterns or constraints, such as ^∗^<worcked> for *worked*; they may also include ambiguous letters, such as writing ^∗^<worced> for *worked.* Finally, when the child’s production has ignored a morpho-graphemic mapping that could resolve an ambiguity, such as ^∗^<workt>for *worked*, the error is classified as morphological. Morphological errors are evaluated on both inflectional and derivational morphemes, as well as in word roots (Bahr et al., 2012). Besides the classification of spelling errors under these three categories, the POMAS allows further specifications within categories, identifying specific mistakes of each kind. In the examples above, <borked> would be further classified as a problem with an initial obstruent (Silliman et al., 2006, p. 111). In the case of <worced>, it would be classified as an error that uses an ambiguous letter: both <c> and <k> can represent /k/, as in *cattle* and *kettle*. The case of <workt> is a morphological error that would be further classified as a problem with (past tense) inflections (Bahr et al., 2012, p. 22).

One of the advantages of a framework such as the POMAS is that it allows for a comprehensive analysis of both the phonological and non-phonological strategies that children use for spelling. Another advantage is that it allows using fine-grained analysis criteria as much (or as little) as the research requires. For these reasons, the POMAS should be helpful in accumulating data from different languages, given that the definition of the main categories should be applicable and comparable cross-linguistically, while the specific phenomena evaluated within each category may be adjusted to each language.

### Adapting POMAS to Assess Catalan Spelling

In this study, we made two adaptations to the POMAS scheme to suit the specific characteristics of Catalan, as well as to answer specific research questions. Catalan is a Romance language spoken in the region of Catalonia, Spain, where the vast majority of people are speakers of, at least, Spanish and Catalan. Catalan is also the language of instruction throughout preschool, primary, and secondary education. It is also used in most university education. Catalan’s morphosyntax is similar to Spanish, in that they both have a rich morphological system, particularly in verbs, and relatively free word order with subject elision (*pro-drop* nature, Bel, 2003). Syllable structure allows, like Spanish, only up to two consonants in onset position, and it is slightly more complex in coda position than Spanish, allowing up to three consonant sounds, as in *boscs/*bosks/“forests.” In the region where the data were collected, Barcelona, Catalan uses eight vowel sounds (three more than Spanish) and has vowel-reduction processes, such that in unstressed syllable position only three vowel sounds may occur: /ə, i, u/(Prieto, 2004). This leads to several spelling ambiguities, because there are multiple possible representations for each of these three phonemes. In addition, some consonants are silent: <h> is always silent, <t> is silent after a nasal sound at the end of words, as in *vent*, “wind”; *caminant*, “walking”; and *estudiant*, “student.” The letter <r> is also silent when word final, as in *primer*, “first,” or *cantar*, “sing.” Several consonant sounds may be represented by two or more graphemes. For example, /b/may be written as <b> or <v>, as in *vaixell*, “boat,” and *bèstia*, “beast”; likewise, the palatal, fricative, voiced sound/ʒ/may be represented by <g>, as in *albergínia*, “aubergine,” and as <j>, as in *jove*, “young.” Some of these inconsistencies can be resolved by applying morphological strategies; for example, the silent <t> in *caminant* indicates that the word is a verb in gerund form. Therefore, if the speller is aware of the link between this morpheme, /-an/, and its spelling, <-ant>, the ambiguity disappears. Other inconsistencies can be resolved by applying orthographic knowledge, that is, knowledge of orthographic patterns and constraints. For example, the use of <g> or <j> to represent phoneme /ʒ/ can be disambiguated by taking into account the following vowel sound: front vowel sounds /e, i, ε/ combine only with <g>, while all other vowel sounds, combine only with <j>. Thus, the correct spelling of /ʒ/ in *albergínia* and *jove* requires accessing and applying knowledge of orthographic patterns. Finally, certain words or parts of words require a full orthographic representation that, often, may need to be paired with a semantic representation. As such, they require memorizing word-specific spellings, since they are not governed by a morphological or an orthographic rule, and phonographic knowledge is necessary but insufficient to arrive at the conventional spelling. This is the case of <v> in *vaixell*, of <b> in *bèstia*, the first <a> in *cantar*, and many others.

Previous studies using POMAS collapsed irregular or word-specific spelling knowledge together with knowledge of orthographic patterns into a single category of “orthographic knowledge” (e.g., Silliman et al., 2006; Bahr et al., 2012). A first adaptation of the present study is, thus, to differentiate between these two types of knowledge sources. We will classify as “orthographic” those errors in which the ambiguity in the representation of a phoneme can be resolved by resorting to knowledge of legal orthographic patterns, and of context-dependent rules. We will classify errors as “lexical” when the ambiguity in the representation of a phoneme cannot be resolved by resorting to rules that can be generalized to several tokens and, rather, the speller needs to resort to word-specific spelling patterns. Often, this will involve an association between the semantics or the lexical representation of a word and its spelling. For example, /hi:l/ associated with the meaning, “to become healthy,” is spelled <heal>, while associated with the meaning “back part of the human foot” is spelled <heel>. A similar distinction was used in another study, also on Catalan (Llauradó and Tolchinsky, 2016). In short, our adaptation entails that the “orthographic” category in POMAS is subdivided into “orthographic” and “lexical” strategies, where the former are characterized by context-dependent rules and knowledge of legal sequences of letters, whereas the latter require rote memorization of words or parts of words, with or without association to a semantic representation.

Our second adaptation of POMAS in the present study is the addition of a new category of *morphophonological* knowledge. This category has been proposed in previous studies of spelling in Dutch and Hebrew (Gillis and Ravid, 2006). Morphophonological representations occur in languages that have productive phonological processes that are triggered as a result of a morphological change. In Catalan, some words include a final occlusive phoneme that is dropped from pronunciation (i.e., it has a zero-realization allophonic variant) only in the case of masculine, singular nouns and adjectives, but that *is* pronounced in other forms of the word. For example, *vent* “wind” is pronounced [ben] in its masculine, singular form, although it is spelled with a silent <t>. In other, usually derived, forms of the word, such as *ventós*, “windy,” or *ventet*, “wind.small,” the <t> is pronounced. This means that the phonological representation of the masculine, singular word must carry the final stop phoneme, /bent/, which is dropped only in this version of the word, but that resurfaces in every other word form. This category entails both access to phonological representations, in combination with knowledge of morphological processes; thus, because it involves the recruitment of strategies beyond phonology, we included it in the present study.

### Previous Research on Spelling Strategies

Some studies using POMAS or evaluating both phonological and non-phonological spelling strategies have examined the relative difficulty of applying them. In general, all strategy types are used even by the youngest participants (e.g., first graders), but there are differences in the rate and developmental route for each type of strategy. Orthographic errors, such as <worcked> or <worced> for *worked*, predominate across grades, while morphological and phonographic errors are the least frequent (e.g., Bahr et al., 2012; Benson-Goldberg, 2014; Llauradó and Tolchinsky, 2016; Joye, 2019). Llauradó and Tolchinsky (2016), who analyzed the spelling errors of 225 Catalan-speaking children in grades 1–5, found that, from grade 2 onward, orthographic (<worcked>) and lexical (<worced>) mistakes were the most frequent and were produced at a similar rate, while they differed significantly from both phonographic (<borked>) and morphological (<workt>) mistakes, which were the least frequent and did not differ from each other. A recent study that developed a pseudo-word spelling task based on triple word-from theory reported that phonographic strategies showed little variation over time, while there were significant differences across grades for orthographic and morphological errors, which decreased over time. This general pattern, according to which error rate is highest for orthographic errors, and lowest for morphological and, especially, phonographic errors, has received support from cross-linguistic studies (e.g., Joye, 2019) and from investigations comparing dyslexic to typically developing controls (e.g., Baseki et al., 2016).

A common trait of previous research using POMAS is that most studies analyzed naturalistic or semi-naturalistic text production data (e.g., Bahr et al., 2012, 2015; Llauradó and Tolchinsky, 2016). While ecologically valid, this procedure has the disadvantage that children may choose less complex or more familiar words (Graham and Harris, 2005), and a different number of opportunities may be created for each type of strategy to be applied. In addition, analyzing misspellings in such a context also poses the challenge of having to determine whether one or more categories apply to a specific error (Bahr et al., 2012, p. 8). For this reason, this study used a bespoke dictation task, in which an equal number of opportunities were created for using each of the non-phonological strategies, while the internal characteristics of the word items (frequency, syllabic complexity, length) were counterbalanced.

To the best of our knowledge, previous research using POMAS has not been used to determine the relative contribution of each type of knowledge source or strategy (i.e., phonographic, orthographic, morphological) to conventional spelling. This is an important point, not only because spelling is paramount to writing quality (e.g., Berninger and Winn, 2006) but also because conventional spelling is the most sensitive measure to predict later literacy gains (Treiman et al., 2019). Arguably, then, understanding the impact that different types of strategies have on children’s spelling skills should be instrumental in determining their importance across a key developmental stage (beginning and intermediate elementary grades) and to orient teaching practices into which strategies require the most attention from practitioners.

### This Study

The present study improves on previous ones in a number of ways. First, it uses a controlled elicitation procedure, a dictation test, instead of naturalistic or semi-naturalistic text production, so that an equal number of opportunities for using each type of strategy were created. In addition, our test measure allowed us to counterbalance word frequency and complexity. Second, it distinguished between rule-bound non-phonological strategies from those that involve memorizing word-specific patterns, not generalizable to other words. Third, in order to truly assess the contribution of non-phonological spelling strategies to spelling development, all analyses were carried out controlling for each child’s phonological accuracy skills. We also included some key demographic variables, sex and parents’ socioeconomic status (SES), as control variables, so as to determine their potential effect (Allred, 1990; Aram and Levin, 2001) and examine the unique contribution of spelling skills and strategies. Fourth, we investigated a relatively unexplored language, Catalan, which has a spelling system much more consistent than English, although it is not exempt from complexities. Finally, we examined spelling-strategy use as a function of children’s exposure to the language of instruction, in order to contribute to the field of second-language spelling and, more specifically, to the development and teaching of spelling in different learning contexts.

The study was articulated around three main research questions: (RQ1) What is the relative difficulty in the application of non-phonological spelling strategies? We hypothesized that morphophonological strategies would be easiest, because they require accessing a productive process that is triggered very frequently both in speech and spelling. We also expected morphological strategies to be among the easier ones, given that previous studies found that morphological errors were the least or second least frequent error type across grades (Bahr et al., 2012; Llauradó and Tolchinsky, 2016). In addition, it has been suggested that speaking a morphologically rich language, as is the case of Catalan, would facilitate the use of morphological strategies (Gillis and Ravid, 2006). In contrast, we expected that the application of context-dependent rules (i.e., orthographic strategies) would be harder, because their overall incidence is lower than both morphological and morphophonological processes. Finally, we expected that word-specific spellings (i.e., lexical strategies) would be the type of strategy that would be used less successfully across grades and population types, given that they are, by definition, only learned through rote memorization.

Our second research question was (RQ2), what is the relative contribution of non-phonological spelling strategies to conventional spelling scores? In this sense, we expected that all strategy types would have a significant contribution, in line with modern views of spelling development that pose that children are sensitive to various sources of knowledge from early on (e.g., Treiman, 2017).

Our third research question was (RQ3), does the use of non-phonological spelling strategies differ for L2 learners or as a function of children’s educational level? With regard to L2 spelling, we expected that children with no exposure to Catalan outside of school would have poorer linguistic and orthographic representations and, therefore, would show more difficulty across all categories. Although the available evidence comparing L1 to L2 spelling is conflicting, we sided with previous literature that poses that, despite differences in performance, a similar pattern of spelling mechanisms underlies both L1 and L2 spelling (e.g., Geva et al., 1993; Verhoeven, 2000). Given that both linguistic and orthographic representations would be less strong in L2 than in L1 children, no single strategy type stood out as more problematic for our L2 participants. Finally, we expected that fourth graders would use all non-phonological spelling strategies more successfully than second graders, but that a similar pattern of difficulty would emerge in both grade levels, in light of previous studies on a similar population (Llauradó and Tolchinsky, 2016).

## Materials and Methods

### Participants

Students were 982 children (497 boys), attending second (494) and fourth (488) grade at schools in the province of Barcelona (Spain). All students were speakers of Catalan, and they were all assumed to be bilingual. Barcelona is a bilingual community, where Catalan is the main language of instruction in elementary education. All students were administered a sociolinguistic questionnaire, which they completed with the help of their teachers. We were particularly interested in the extent to which children used Catalan outside school, so they were asked to declare the language(s) they used with each of their parents, with siblings, and with friends. Based on this information, we classified children as being exposed to Catalan outside of school or not. Table 1 provides the demographic information and distribution of the sample.

**TABLE 1:** Participants’ distribution and demographic information.

Group	Number of participants	Mean age (SD)	Boys/girls	Mean SES (SD)
Cat0-G2	238	7;5 (0;4)	113/125	41.24 (11.91)
Cat0-G4	214	9;3 (0;8)	118/96	39.70 (10.50)
Cat1-G2	256	7;4 (0;4)	125/131	52.10 (15.38)
Cat1-G4	274	9;4 (0;4)	141/133	53.05 (14.59)

*G2, grade 2; G4, grade 4; Cat0, no Catalan exposure outside school; Cat1, some degree of Catalan exposure outside school (e.g., child speaks Catalan with mother).*

The study belongs to a larger project on writing development that had obtained full clearance by the Ethics Committee of the University. Children were recruited from 13 public schools. All children in the classroom were approached, and we collected data only from those whose parents returned signed consent forms. No children were excluded from the study on the basis of learning disorders.

### Task Design and Coding

All the children completed the same bespoke task, which involved spelling 34 words that were dictated orally by the administrator. Only 32 items were used here, as two items targeted stress-mark spelling, which was outside the scope of the present study. The items were selected from a corpus of Catalan children’s spelling (Llaurado et al., 2012), available online at http://clic.ub.edu/corpus/en/cesca-en. This corpus includes the words that a sample of more than 2000 children aged 5–16 wrote in response to a semantic category (e.g., clothes, traits of character, natural phenomena, food). Words are lemmatized and can be looked up according to their overall frequency, the educational level of the writer, and they are moreover listed according to the number of alternative (mis)spellings produced. Words that appeared in the 90th percentile or higher were considered high-frequency words, and words in the 10th percentile or lower were considered low-frequency words, as long as they belonged to the corresponding category within which they were produced^1^.

The task was designed as follows: 32 items were selected so that at least one phoneme was ambiguous (i.e., it had two or more alternative spellings). A single phoneme was targeted in each item, whose ambiguity could be eliminated by resorting to one of four different sources of knowledge: morphological, morphophonological, orthographic, or lexical. There were eight items per category. In addition, all items were scored as correct or incorrect in terms of their phonographic plausibility, that is, whether the spelling was an accurate phonological representation of the intended word, regardless of positional or other orthographic constraints. Finally, items were scored as correct or incorrect in terms of conventional accuracy (i.e., as they would appear in a dictionary). Items were counterbalanced for frequency: 16 low-frequency items, 16 high-frequency items; for length, with *short* words consisting of one or two syllables and *long* words consisting of three to four syllables; and for syllabic complexity: *simple* words typically consisting of open syllables, usually CV in structure, while *complex* words had, at least, two closed (e.g., CVC) syllables or at least one consonant cluster (e.g., CCV, VCC). All items were validated by two experienced elementary-school teachers, who confirmed the perceptions of frequency and difficulty.

In the morphophonological category, all eight items had a silent <t> that could be recovered in derived words. In the morphological category, four items tested the silent <t> in the gerund form of verbs, and the other four items tested the spelling of the plural of feminine nouns, which contains an ambiguous vowel sound, /ə/, that is always spelled <e>. In the orthographic category, seven items tested <g> vs. <j> alternation, which is regulated by the following vowel, and one item tested knowledge that the spelling of the sound /z/ after a consonant sound is always <z>. Finally, the lexical category tested some of the most common homophonous letters, when they are not governed by other rules: <b, v>, <h, ->, <a, e>, <s, ss, ç>.

Several scores were obtained from the task: (1) a conventional spelling accuracy score, (2) phonographic plausibility, (3) use of morphological strategies, (4) use of morphophonological strategies, (5) use of orthographic strategies, and (6) use of lexical knowledge. Each score was determined independently of the rest, in a binary fashion. Conventional accuracy and phonological plausibility scores were determined for all 32-word items, whereas for each type of non-phonological strategy, the score was the total correct out of the eight items in the category. Cronbach alpha reliability for the test was 0.92. An external research assistant, uninvolved in the present research, scored all words, and the author rescored 28% of a random sample. Inter-rater reliability [intra-class correlation (ICC)] was excellent: 0.989.

### Procedure

Children were tested in their regular classrooms. They were given a lined paper, with a dot next to which they had to write each word, one below the other. The administrator explained that they were going to do a dictation task and that they would hear each word three times: first in isolation, then in a carrier sentence, which would help identify the word used in context. Finally, they would hear it one final time, again in isolation. For example, the administrator said, “*Vent. Si fa vent, podrem anar a navegar. Vent.*” “Wind. If there’s wind, we can go sailing. Wind.” Children were given a few seconds to spell each word. They were encouraged to write them as best they could, even if they were unsure, and told not to worry if they did not know how to spell correctly. Testing lasted between 20 and 30 min, a time that all teachers reported they were used to engaging in writing activities.

All test administrators received training as to how to deliver the sentences in terms of speed, rhythm, and emphasis. They practiced several times until it was clear that they were all reading the items using a similar, natural tone, and without specifically emphasizing any of the words.

## Results

An inspection of the data showed that they were normally distributed, with skewness values below 3, and kurtosis values under 10 (Kline, 2011). We conducted a series of analyses dividing children into four groups: grade 2 without Catalan exposure, grade 2 with Catalan exposure, and analogous grade 4 groups.

### Development of Non-phonological Spelling Strategies

We first addressed the difficulty of applying the various non-phonological spelling strategies in each group. We ran preliminary one-way, repeated-measures ANOVAs to determine whether the type of spelling strategy that needed to be applied in each group of eight words affected performance. Results showed that the type of strategy did have a significant effect on its successful application, with moderate effect sizes across groups (Table 2). In general, application of a morphophonological or an orthographic strategy was easier, whereas lexical and morphological strategies were harder strategies across groups. It was apparent, however, that there were differences between groups.

**TABLE 2:** Comparison between performance measurements of different word types by grade level and exposure to Catalan.

Group	Lexical	Morph	MPhon	Ortho	df	*F*	$\eta_{\text{p}}^{2}$
G2 Cat0	0.35^a^ (0.23)	0.31^a^ (0.29)	0.53^c^ (0.29)	0.42^b^ (0.35)	3633	44.32***	0.17
G2 Cat1	0.32^a^ (0.22)	0.35^a^ (0.28)	0.55^b^ (0.30)	0.56^b^ (0.32)	3726	79.77***	0.25
G4 Cat0	0.51^a^ (0.22)	0.48^a^ (0.29)	0.74^c^ (0.24)	0.62^b^ (0.31)	3579	72.12***	0.27
G4 Cat1	0.53^a^ (0.22)	0.55^a^ (0.28)	0.78^c^ (0.23)	0.67^b^ (0.28)	3783	96.98***	0.27

G2, grade 2; G4, grade 4; Cat0, no Catalan exposure outside school; Cat1, some degree of Catalan exposure outside school (e.g., child speaks Catalan with mother); Ortho, proportion of correct use of orthographic knowledge; MPhon, proportion of correct use of morphophonological knowledge; Morph, proportion of correct use of morphological knowledge; Lex, proportion of correct use of lexical knowledge; Small Latin letters for mean ranking, from the lowest (a) to the highest. ***p < 0.001.

We next conducted a more comprehensive two-level structural-equation model (SEM), in which students were repeatedly measured on the four types of words. Thus, the level 1 data were the item-level performance and the level 2 data were the students. Moreover, this modeling strategy was run within a multiple-group comparison framework, where we used the division of children into the four groups specified above and tested the effect of Strategy Type on performance. The model included sex, parents’ SES, and each child’s phonographic accuracy score as control variables. The overall unconstrained model goodness of fit was excellent, *Chi*^2^(8) = 6.96, *p* = 0.541; RMSEA = 0.000; CFI = 1.00; TLI = 1.00; SRMR_within_ = 0.000; and SRMR_between_ = 0.019. In each group, we were able to show pairwise comparisons between effects (Table 3). For both grades 2 and 4 with no Catalan outside of school, the order of difficulty was the same reported in the ANOVAs (from easiest to hardest): morphophonological, orthographic, morphological, and lexical. All comparisons between each pair of strategies were significant across grade levels. In the case of children with exposure to Catalan outside school, there were only subtle variations. For second graders, letters that required a morphophonological or an orthographic strategy were equally difficult; similarly, applying a morphological or lexical strategy was equally hard. These two pairs of strategies, orthographic and morphophonological, on the one hand, and lexical and morphological, on the other, were significantly different from each other. In the fourth grade, the situation was similar, except that these children found that applying a morphophonological strategy was significantly easier than applying an orthographic one.

**TABLE 3:** SEM comparative coefficients between types of strategies.

Group	L-O	M-O	MP-O	L-M	L-MP	M-MP
G2 Cat0	−0.07* (0.02)	−0.10* (0.02)	0.13* (0.03)	0.04* (0.02)	−0.19*** (0.02)	−0.23*** (0.02)
G2 Cat1	−0.27* (0.02)	−0.21* (0.02)	−0.01 (0.02)	−0.03 (0.02)	−0.23*** (0.02)	−0.20*** (0.01)
G4 Cat0	−0.11* (0.02)	−0.14* (0.02)	0.12* (0.02)	0.04* (0.02)	−0.22*** (0.02)	−0.26*** (0.02)
G4 Cat1	−0.14* (0.02)	−0.11* (0.02)	0.12* (0.02)	−0.02 (0.02)	−0.25*** (0.01)	−0.23*** (0.01)

G2, grade 2; G4, grade 4; Cat0, no Catalan exposure outside school; Cat1, some degree of Catalan exposure outside school (e.g., child speaks Catalan with mother); O, proportion of correct use of orthographic knowledge; MP, proportion of correct use of morphophonological knowledge; M, proportion of correct use of morphological knowledge; L, proportion of correct use of lexical knowledge. ***p < 0.001 and *p < 0.05.

As the SEM model was built of two-level data, we estimated the impact of students’ characteristics on performance. The ICCs of the success rate of Strategy Type were above 0.30, which emphasized the potential of variability across students. In all four groups separately, students’ sex and parents’ SES did not have a significant effect on performance. Phonographic accuracy, in contrast, did have a significant effect, so that children with higher scores on phonographic accuracy tended to apply non-phonological spelling strategies more successfully. Adding Strategy Type as an independent variable considerably improved the explanatory power of the model, as shown by the notable increase pseudo *R*^2^ values of the final model vs. the model without level 1 indicators, from a.14 to a.21 increment (Table 4). These findings were consistent across the four groups. Students’ demographic characteristics did not impact their performance, while complementary indicators did.

**TABLE 4:** Structural equation modeling results for level 2 student’s effects by grade and exposure to Catalan.

			Unconditional model		Conditional model with no strategy type			Final model				
					
Group	Sex	SES	Phono. Acc.	ICC	Within variance	Between variance	Within variance	Between variance	Pseudo *R*^2^	Within variance	Between variance	Pseudo *R*^2^
G2 Cat0	0.03 (0.02)	−0.002 (0.001)	0.84*** (0.06)	0.383	0.057 (0.003)	0.035 (0.005)	0.056 (0.003)	0.013 (0.003)	0.26	0.046 (0.003)	0.015 (0.003)	0.40
G2 Cat1	−0.02 (0.02)	0.000 (0.001)	0.77*** (0.06)	0.297	0.065 (0.003)	0.027 (0.004)	0.064 (0.003)	0.010 (0.002)	0.20	0.049 (0.002)	0.014 (0.002)	0.38
G4 Cat0	0.01 (0.02)	0.00 (0.001)	0.97*** (0.07)	0.367	0.051 (0.003)	0.030 (0.004)	0.052 (0.003)	0.006 (0.002)	0.28	0.038 (0.003)	0.010 (0.002)	0.49
G4 Cat1	−0.02 (0.02)	0.001 (0.001)	0.93*** (0.06)	0.336	0.050 (0.002)	0.025 (0.004)	0.050 (0.002)	0.004 (0.002)	0.28	0.037 (0.002)	0.008 (0.002)	0.48

G2, grade 2; G4, grade 4; Cat0, no Catalan exposure outside school; Cat1, some degree of Catalan exposure outside school (e.g., child speaks Catalan with mother); SES, socioeconomic status; Phono. Acc., phonological accuracy. ***p < 0.001.

Because there were remarkable similarities across groups in terms of (1) the pattern of relative difficulty for each spelling strategy; (2) the influence of demographic variables; and (3) the role of phonological accuracy, we ran alternative, more parsimonious models in which we constrained either Educational level or Exposure to Catalan to be equal across groups. These models, however, were a significantly worse fit to the data, *Chi*^2^(6) > 30.21, *p* > 0.05, supporting the between-group differences reported above.

### The Contribution of Non-phonological Strategies to Conventional Spelling

A second series of models examined the contribution of non-phonological spelling strategies to explaining conventional accuracy scores. We ran a one-level, multigroup SEM, in which conventional accuracy was the dependent variable and each group was defined, as in previous analyses, according to the educational level (grade 2 or 4) and exposure to Catalan outside school (exposure, no exposure). A baseline model that included sex and SES, as well as phonological accuracy, was an excellent fit to the data *Chi*^2^(8) = 7.10, *p* = 0.530; RMSEA = 0.000; CFI = 1.000; TLI = 1.002; SRMR = 0.027, and explained a significant proportion of the variance, with *R*^2^ values ranging from 0.430 to 0.583. A model in which children’s performance on each type of non-phonological spelling strategy was added as an independent variable was also a great fit to the data, Δ*Chi*^2^(26) = 25.1, *p* > 0.05, and it explained a much larger proportion of variance: *Rs*^2^ = 0.848 and 0.865, for grades 2 and 4 without Catalan exposure outside school, and *Rs*^2^ = 0.841, and 0.866 for grades 2 and 4 with Catalan exposure outside school, respectively. Finally, a model in which the influence of non-phonological spelling strategies on conventional accuracy was constrained to be equal across all four groups was an excellent fit to the data as well, *Chi*^2^(46) = 42.05, *p* = 0.638; RMSEA = 0.000; CFI = 1.000; TLI = 1.001; SRMR = 0.036; Δ*Chi*^2^(12) = 9.95, *p* > 0.05. This final model indicated that, above and beyond the significant effect of phonographic accuracy, conventional accuracy was affected by children’s ability to apply non-phonological strategies. In contrast, children’s sex or their parents’ SES did not exert a substantial influence. Moreover, our results show that morphological and lexical skills contributed more to conventional accuracy scores than either orthographic or morphophonological skills (Table 5).

**TABLE 5:** Unstandardized estimates, standard errors, and significance values of SEM on conventional spelling accuracy scores (final model^†^).

Variable	Estimate^2^	SE	*p*-Value
Lexical^1^	0.320	0.021	<0.001
Morphological^1^	0.326	0.020	<0.001
Morphophonological^1^	0.157	0.020	<0.001
Orthographic^1^	0.180	0.020	<0.001
Phonological accuracy	0.213	0.029	<0.001
Sex	−0.002	0.028	0.087
SES	0.026	0.036	0.232

*^1^Strategy type; ^2^standardized estimates; SE, standard error. ^†^Model in which all groups were constrained to be equal.*

## Discussion

This study set out to examine the role of non-phonological strategies in spelling development. A large number of children in grades 2 and 4, speakers of Catalan, completed a dictation task that included words containing a target inconsistent phoneme. Words were grouped according to the type of non-phonological strategy that was required to resolve an inconsistency: morphological, morphophonological, orthographic, and lexical. In addition, children’s production of each word was assessed in terms of conventional and phonographic accuracy. We aimed to ascertain the relative difficulty of applying different types of non-phonological strategies, and the contribution of each strategy to conventional accuracy scores, above and beyond the effect of children’s phonographic skills.

### Development of Non-phonological Spelling Strategies

Our first research question involved the relative difficulty with which children applied the various non-phonological strategies, controlling for their phonographic spelling skills. Our findings showed that, generally speaking, strategies that required morphophonological or orthographic knowledge were mastered earlier than those requiring morphological or lexical knowledge. These results partially confirmed our initial hypotheses. We had expected morphophonological strategies to be on the easier side of the continuum, given that we hypothesized that even our youngest age group, the second graders, would succeed in efficiently accessing the full phonological representation of words. In addition, the phenomenon that creates the inconsistency, namely, the zero-realization of the final stop phoneme, although triggered by a morphological process, is extremely common in everyday speech. Indeed, children seemed to be able to extend their knowledge of phonological representations to represent silent letters and to take advantage of the phonology–morphology interface, in line with previous studies (Gillis and Ravid, 2006).

We had also expected that the lexical strategy would be among the hardest to apply, given that it involves word-specific knowledge, so children would only be able to produce the correct spelling if they already had an orthographic representation of the word. Our findings for the lexical category supported this hypothesis. This means that, even in semi-consistent orthographies like Catalan, strong orthographic representations are needed to spell words that are not entirely rule-bound (e.g., Carrillo et al., 2013; Marinelli et al., 2017; Angelelli et al., 2018). It is likely that vocabulary knowledge and strong semantic representations are advisable teaching strategies to enhance this source of knowledge to improve children’s spelling skills.

As for orthographic strategies, we had expected that they would be relatively easy to apply across groups. Results supported this hypothesis. Previous studies using POMAS had found this category to be among the least successful (e.g., Bahr et al., 2012; Llauradó and Tolchinsky, 2016; Daffern and Ramful, 2020). However, we expected a different result based on the fact that, in contrast to most previous research using POMAS, we had distinguished between types of misspellings, reserving this category only for spelling mistakes that involved overlooking orthographic patterns and constraints, while word-specific strategies were under the “lexical” category. Given the fact that our orthographic category required applying constraints to which children have been found to be sensitive from early on (Cassar and Treiman, 1997), our expectation was that our participants would readily choose between homophonous letters using orthographic knowledge. It should be noted, however, that one study, also on Catalan, made the same distinction between orthographic and lexical errors (Llauradó and Tolchinsky, 2016) and found orthographic strategies to be harder than phonographic and morphological strategies. We would speculate that the differences with this previous study are due to the fact that Llauradó and Tolchinsky (2016) analyzed words that children had produced in a relatively free writing context, while we analyzed only a specific segment (phoneme) in a closed group of words, targeting a single ambiguous phoneme, /ʒ/. The orthographic rules that need to be applied to choose between homophonous letters in our case are taught at school, and, although children do make substitution mistakes involving <g> and <j>, it is reasonable to think that a lot of them were aware of the rule. Future studies should test a more comprehensive set of orthographic-bound inconsistencies to determine whether orthographic knowledge is indeed easier to apply than other types of strategies or whether it is highly dependent on the particular context chosen.

The most striking finding concerns our results for morphological strategies. We expected children to be quite adept at using morpho-graphemic regularities to resolve very common spelling inconsistencies, such as plural or gerund formation. Our expectation was based on (1) the results from other studies using POMAS, which reported morphological errors to be one of the least frequent (e.g., Bahr et al., 2012; Llauradó and Tolchinsky, 2016; Daffern and Ramful, 2020) and (2) the fact that children are speakers of a morphologically rich language, which is a key factor in determining their sensitivity to morphological information (e.g., Gillis and Ravid, 2006; Dressler, 2010). Nevertheless, in the present study, morphological strategies were one of the hardest to be applied across educational levels and population types. This was a surprising result, especially because we only tested regular inflectional morphology (plural formation and gerunds), which has been reported to be the easiest context for the application of morphological knowledge in spelling (e.g., Llauradó and Tolchinsky, 2016; Daffern and Ramful, 2020). We believe that the differences with past research are essentially methodological. Most previous studies did not control for the occurrences of morphologically bound spellings. Particularly in studies that analyzed free writing samples (e.g., Bahr et al., 2012), each text will have created a different number of opportunities in which application of a morpho-graphemic mapping was relevant (e.g., regular past tenses, derivates, plurals, and so on). Therefore, the low rates of morphological mistakes found in those studies might be merely reflecting instances in which morphological spellings were applicable (and, certainly, not resolved successfully). The current study arguably provides a more reliable result, given that children encountered the exact same number of opportunities for the application of each strategy. Our findings thus indicate that, even in a language with rich morphology like Catalan, children struggle to mobilize this aspect of their linguistic knowledge to use it for spelling. Previous research on morphological awareness indicated that it is, indeed, a protracted development (e.g., Green et al., 2003), although intervention studies to improve it are generally successful (e.g., Devonshire and Fluck, 2010; Devonshire et al., 2013; Bowers and Bowers, 2017). A key educational implication is, thus, that children need to be taught about the way words are formed and how these forms map onto the orthography.

### Contribution of Non-phonological Strategies to Conventional Spelling

A second research question concerned the impact of the various non-phonological spelling strategies on spelling accuracy scores, over and above that of children’s phonographic skills. A major novel outcome of the present study was the finding that all non-phonological strategies had a significant and unique contribution to spelling conventionally across educational levels and population types. In this way, we have provided substantial support to theories that pose that learning to spell requires mobilizing various types of linguistic knowledge besides phonological skills (e.g., Rittle-Johnson and Siegler, 1999; Bahr et al., 2009; Treiman, 2017). Learning to spell involves phonological skills and knowledge of letter-sound correspondences (e.g., Caravolas et al., 2012), but it also requires knowledge of orthographic constraints, of morpho-graphemic regularities, and word-specific orthographic representations of tokens whose spelling cannot be ascertained by generalizable patterns.

The fact that the impact of non-phonological spelling strategies on conventional spelling accuracy was unaffected by children’s educational level indicates that these various types of knowledge sources are operative over a wide developmental span. Similarly, the lack of an educational-level effect questions phonology-first approaches to literacy development [e.g., National Reading Panel, 2000] and calls for further research on the importance of mobilizing all levels of linguistic representations relevant for spelling from the earlier grades.

These findings are in line with previous studies claiming that non-phonological spelling strategies are necessary also in orthographies more transparent than English or French (e.g., Lehtonen and Bryant, 2005; Defior et al., 2008; Carrillo and Alegría, 2014; Rothe et al., 2014; Angelelli et al., 2017, 2018). It could be argued that users of orthographies with very consistent phoneme-to-grapheme mappings would rely to a great extent on these simple associations, rather than apply a host of different strategies to spell accurately. However, spellers appear to take advantage of additional knowledge sources that may help to disambiguate between alternative, homophonous spellings regardless, in principle, of how inconsistent the system is. Future research should strive to compare efficacy in strategy use (both phonological and non-phonological) across languages.

### Use of Non-phonological Strategies and Grade

As expected, fourth graders outscored second graders in all strategy types, but the general pattern of difficulty applied across grades: morphophonological and orthographic strategies were generally easier to apply than both morphological and lexical strategies. These findings contradict stage-like views of spelling that consider non-phonological strategies a later development (e.g., Frith, 1985). On the contrary, the present study provides further support to spelling development theories that claim that non-phonological spelling strategies are used from very early on (e.g., Rittle-Johnson and Siegler, 1999; Treiman, 2017).

The current study also has clear educational implications. On the one hand, since all non-phonological strategies are paramount for spelling accurately, the teaching of spelling should strive to mobilize all relevant linguistic levels from very early on (e.g., Devonshire et al., 2013). On the other hand, at least from grade 2 onward, teachers should target lexical and morphological strategies in particular, in an overall multidimensional approach to spelling instruction. This is because it was precisely these strategies that not only proved to be the hardest but also were the ones that made the largest contribution to spelling conventionally. Morphological strategies could be taught by raising children’s levels of morphological awareness, explicitly teaching them about word formation, while showing the specific (and consistent) way in which morphemes map onto the orthography (Alves et al., 2018). Lexical strategies should be promoted by calling children’s attention to word-specific spellings in meaningful contexts, with the overarching goal of facilitating the creation of robust orthographic representations filled with semantic information (Treiman and Kessler, 2014; Treiman, 2017).

### L1 and L2 Non-phonological Spelling Strategies

Second-language learners who do not have at-home support of the language of instruction found it harder to apply all spelling strategies, as expected. However, they did not show a unique pattern of strategy use and, just as the L1 participants, found it easier to apply morphophonological and orthographic knowledge, than to apply morphological or lexical knowledge. Despite not showing a unique developmental route, we did not find support for a common model of spelling strategy use (that is, one that did not distinguish between these two population types), suggesting that performance differences were substantial, in line with previous studies (e.g., Geva et al., 1993; Verhoeven, 2000). Notably, this was true for children in grade 2 as for children in grade 4, indicating that having little or no contact with the language of instruction outside school has a long-lasting impact on these children’s literacy development. This means that L2 learners require extra support for spelling development beyond phonology (e.g., Bar-Kochva and Hasselhorn, 2017; Bowers and Bowers, 2017), though such training can be similar in nature to training aimed to their L1 peers (e.g., Devonshire and Fluck, 2010; Devonshire et al., 2013; Alves et al., 2018). Without adequate support, however, these children could be at risk of academic failure, in view of the key role that spelling has on writing development (e.g., Juel, 1988; Salas and Silvente, 2019).

The similarities found across L1 and L2 spelling strategies were even clearer in the contribution of the various non-phonological strategies to conventional spelling, above and beyond the contribution of phonographic strategies and demographic factors. Results were consistent with studies reporting that L1 and L2 spelling have similar drivers of performance (Geva et al., 1993; Verhoeven, 2000), given that all non-phonological strategies contributed greatly to explaining conventional spelling accuracy across population types. The lack of qualitative differences in the underpinnings of spelling development as a function of language status (i.e., L1 vs. L2) extends previous assertions about potentially universal factors driving (early) literacy development, at least in alphabetic writing systems (Caravolas et al., 2012).

### Limitations

As with virtually every other developmental study, it would have been ideal to test the same hypotheses on longitudinal, rather than cross-sectional data. Future studies should try to inquire whether the same pattern of results is replicated in longitudinal datasets, particularly encompassing a larger developmental span. Another shortcoming of the present study is that it included a limited number of use cases within each strategy type. This affects particularly the application of orthographic knowledge, in view that some orthographic patterns might be more difficult to learn than others. Similarly, research on morphological spelling strategies would benefit from investigations that include a larger set of contexts, with not just inflectional but also derivational morpho-graphemic correspondences. Finally, the present findings may only be applicable to the language under examination, Catalan, although they can be accumulated to previous studies that used a similar error-analysis approach. Cross-linguistic studies should be able to shed light on whether the trends presented are part of a language-general pattern of spelling development.

## Conclusion

Spelling is a multidimensional skill that involves phonographic knowledge, but to which non-phonological strategies make a large contribution early on in development. We have shown that, even in a relatively consistent orthography, strategies beyond phonology that involve morphophonological, morphological, orthographic, and lexical knowledge are instrumental to spell accurately. In particular, knowledge of morpho-graphemic correspondences and word-specific orthographic representations made the largest contribution to conventional spelling scores in early and intermediate elementary school levels. L2 learners had more difficulty to apply all strategy types than peers who have target language exposure outside school, but they seem to follow the same developmental route. Across population types and regardless of children’s sex or their parents’ SES, applying morphological and lexical strategies was more challenging than applying morphophonological and orthographic regularities. Spelling instruction should therefore strive to adopt a multidimensional approach from the earlier grades and provide extra support to children without exposure to the language of instruction outside school.

## Data Availability Statement

The datasets generated for this study are available on request to the corresponding author.

## Ethics Statement

The studies involving human participants were reviewed and approved by Comissió d’Ètica en l’Experimentació Animal i Humana, UAB. Written informed consent to participate in this study was provided by the participants’ legal guardian/next of kin.

## Author Contributions

NS designed and conducted the study, analyzed the spelling samples, carried out statistical analyses, and wrote the manuscript in its entirety.

## Conflict of Interest

The author declares that the research was conducted in the absence of any commercial or financial relationships that could be construed as a potential conflict of interest.

## Footnotes

1 Sometimes children would produce a word in response to, for example, traits of character, that had an overall low frequency, such as *beautiful*. These instances were ignored, as the resulting word may not necessarily be a low-frequency token in absolute terms. Rather, they seem to be productions that failed to capture the semantic field that was required.
